# Monoclonal antibodies against S2 subunit of spike protein exhibit broad reactivity toward SARS-CoV-2 variants

**DOI:** 10.1186/s12929-022-00891-2

**Published:** 2022-12-22

**Authors:** Shih-Han Ko, Wan-Yu Chen, Shih-Chieh Su, Hsiu-Ting Lin, Feng-Yi Ke, Kang-Hao Liang, Fu-Fei Hsu, Monika Kumari, Chi-Yu Fu, Han-Chung Wu

**Affiliations:** 1 Biomedical Translation Research Center (BioTReC), Academia Sinica, Taipei, 11529 Taiwan; 2 Institute of Cellular and Organismic Biology, Academia Sinica, Taipei, 11529 Taiwan

**Keywords:** Severe acute respiratory syndrome coronavirus 2 (SARS-CoV-2), Spike (S) protein, Monoclonal antibody, Phage display, B cell epitope

## Abstract

**Background:**

The variants of severe acute respiratory syndrome coronavirus 2 (SARS-CoV-2) harbor diverse spike (S) protein sequences, which can greatly influence the efficacies of therapeutics. Therefore, it would be of great value to develop neutralizing monoclonal antibodies (mAbs) that can broadly recognize multiple variants.

**Methods:**

Using an mRNA-LNP immunization strategy, we generated several mAbs that specifically target the conserved S2 subunit of SARS-CoV-2 (B-S2-mAbs). These mAbs were assessed for their neutralizing activity with pseudotyped viruses and binding ability for SARS-CoV-2 variants.

**Results:**

Among these mAbs, five exhibited strong neutralizing ability toward the Gamma variant and also recognized viral S proteins from the Wuhan, Alpha, Beta, Gamma, Delta and Omicron (BA.1, BA.2 and BA.5) variants. Furthermore, we demonstrated the broad reactivities of these B-S2-mAbs in several different applications, including immunosorbent, immunofluorescence and immunoblotting assays. In particular, B-S2-mAb-2 exhibited potent neutralization of Gamma variant (IC_50_ = 0.048 µg/ml) in a pseudovirus neutralization assay. The neutralizing epitope of B-S2-mAb-2 was identified by phage display as amino acid residues 1146–1152 (DSFKEEL) in the S2 subunit HR2 domain of SARS-CoV-2.

**Conclusion:**

Since there are not many mAbs that can bind the S2 subunit of SARS-CoV-2 variants, our set of B-S2-mAbs may provide important materials for basic research and potential clinical applications. Importantly, our study results demonstrate that the viral S2 subunit can be targeted for the production of cross-reactive antibodies, which may be used for coronavirus detection and neutralization.

**Supplementary Information:**

The online version contains supplementary material available at 10.1186/s12929-022-00891-2.

## Background

Since the end of 2019, severe acute respiratory syndrome coronavirus 2 (SARS-CoV-2) has spread throughout the world in an immunologically naïve population, causing significant morbidity and mortality [1]. As SARS-CoV-2 continues to evolve through mutations in its viral genomic RNA, viral variants of concern periodically emerge and pose new threats to public health [2]. The SARS-CoV-2 Omicron variants were first detected in November 2021 and quickly outcompeted other variants, such as Alpha (B.1.1.7), Beta (B.1.351), Gamma (P.1), Delta (B.1.617), due to their especially high transmissibility and immune evasion [3]. Since its emergence, the initial Omicron variant BA.1 has split into several other sublineages, including BA.2, BA.3, BA.4 and BA.5. Notably, all of these Omicron variants carry multiple mutations in the viral spike (S) protein that significantly reduce vaccine efficacies and markedly raise the risk of breakthrough infections [4]. The SARS-CoV-2 S protein is composed of S1 and S2 subunits, which adopt a trimeric conformation with three receptor-targeting S1 heads and an S2-trimer stalk on the surface of the virus particle [5]. The BA.1 sublineage of Omicron harbors 28 altered amino acid residues in the S1 subunit and 6 altered amino acid residues in the S2 subunit compared to the original Wuhan strain [6]. In contrast to the frequently mutated S1, the S2 subunit is more conserved among SARS-CoV-2 variants [6, 7].

When SARS-CoV-2 infects cells, the viral S protein binds to human angiotensin converting enzyme 2 (hACE2) and is proteolytically activated by human proteases, furin and TMPRSS2, which respectively cleave the S1/S2 and the S2’ sites of the S protein to cause dissociation the S1 and S2 subunits [8–10]. The functional domains of the S2 subunit are known to include an N-terminal hydrophobic fusion peptide (FP), heptad repeats- HR1 and HR2, a transmembrane domain (TM), and a cytoplasmic tail [11]. The S2 subunit changes from its pre-fusion conformation to a post-fusion structure following the interaction between the S1 receptor-binding domain (RBD) and the hACE2 receptor. The post-fusion conformation of S2 comprises a six-helix bundle fusion core structure that allows insertion into the host cell membrane, along with two HR domains that promote viral fusion by reducing the distance between the cell membrane and viral envelope [12]. A previous study on SARS-CoV showed that peptides derived from HR2 may bind to the HR1 domain of the S2 subunit and prevent the conformational shift of S protein, blocking viral fusion and entry [13]. Similarly, another group of researchers generated a recombinant protein SARS-CoV-2 fusion inhibitor interacts with HR2 to block formation of the six-helix bundle fusion core [14]. In addition, human antibodies against the S2 subunit that were isolated from COVID-19 convalescent patients were found to exhibit neutralizing activity for multiple betacoronaviruses [15–18]. Together, these studies suggest that the S2 subunit of SARS-CoV-2 may be a promising target for novel therapeutics.

Recently, intramuscular (I.M.) injection of mRNA-containing lipid nanoparticles (mRNA-LNPs) has been used to stimulate the production of neutralizing antibodies against COVID-19, particularly with regard to the Moderna and Pfizer-BioNTech vaccines [19]. Our group previously showed that mRNA-LNP immunization is also an effective strategy to stimulate the production of specific monoclonal antibodies (mAbs) in mice. In that study, we used a viral RBD mRNA-LNP immunogen to stimulate immune response and isolated mAbs against the viral RBD that exhibited broad neutralizing activity for several SARS-CoV-2 variants [20, 21]. Since the S2 subunit is generally conserved across SARS-CoV-2 variants and other betacoronaviurses [15], we sought to generate mAbs that specifically target the S2 subunit using a mRNA-LNP containing viral S mRNA of SARS-CoV-2 Beta variant. We then selected five B-S2-mAbs that cross-reacted with the S proteins of multiple SARS-CoV-2 variants and further exhibited neutralizing activity against pseudotyped SARS-CoV-2. One of the B-S2-mAbs was found to target a specific site in the S2 subunit HR2 domain, which is highly conserved during viral evolution. These B-S2-mAbs were then used for recognition of SARS-CoV-2 variants in a variety of applications.

## Methods

### Generation of mAbs against S2 subunit of S protein using mRNA-LNP immunization

The Beta variant S mRNA was produced by in vitro transcription using T7 polymerase (NEB; E2050S) and EcoRV-linearized S vector that contained 5′-UTR, signal peptide from Igκ, 3′-UTR and poly-A tail. CleanCap^®^AG (Trilink; N-7413) was used for mRNA capping in a Cap 1 structure; uridine was replaced with N1-methyl-pseudouridine (Trilink; N-1081) [21]. The preparation and characterization of mRNA-LNPs were performed as described previously [21, 22]. Briefly, the LNP consisted of four types of lipids, including D-Lin-MC3-DMA (MedChemExpress; HY-112,251), DSPC (1,2-distearoyl-sn-glycero-3-phosphocholine; Avanti; 850365P), cholesterol (Sigma-Aldrich; C3045) and DMG-PEG 2000 (MedChemExpress; HY-112,764) at ratios of 50:10:38.5:1.5. Preparation of mRNA-LNP by mixing lipid and beta variant S mRNA in a 3:1 ratio using NanoAssemblr® Ignite™ (Precision NanoSystems). The mRNA-LNPs were prepared by mixing beta variant S mRNA and lipids mixture at a flow rate ratio of 3:1 by using NanoAssemblr®Ignite™ (Precision NanoSystems). The mRNA-LNPs were then dialyzed with phosphate-buffered saline (PBS). Finally, Zetasizer Nano ZS was used to measure the size and zeta potential of the mRNA-LNP (Malvern Panalytical Ltd.). For immunization, female BALB/c mice (6–8 weeks of age) were immunized with 10 µg of mRNA-LNP encoding the Beta variant S protein (1-1255 amino acids) by I.M. injection every 2 weeks. After the third immunization, the antibody titer against S protein (Beta strain; ACRO Biosystems, SPN-C52Hc) was evaluated by an immunosorbent assay. A booster dose of 25 µg recombinant S protein (Beta strain; ACRO Biosystems, SPN-C52Hc) was administered by intraperitoneal injection at week 7, after which mouse myeloma NS1 cells were fused with mouse splenocytes for selection and generation of mAbs. All animal experiments were approved by the Academia Sinica Institutional Animal Care and Use Committee (IACUC protocol No. 20051468).

### VSV-based pseudovirus neutralization assays

SARS-CoV-2 pseudovirus neutralization assays were performed as previously described [23, 24]. Briefly, human ACE2-expressing HEK293T (hACE2-HEK293T) cells were infected with pseudotyped SARS-CoV-2 virus including VSV envelope glycoprotein bearing a luciferase gene as a reporter; the virus was provided by the National RNAi Core Facility (Academia Sinica, Taiwan). Serial dilutions of B-S2-mAbs were mixed with pseudovirus bearing S protein of SARS-CoV-2 variants (1000 TU) in DMEM (Gibco; 12100-046) containing 1% FBS (Gibco; 10437-028) for 1 h at 37 °C. The mixtures were then applied to hACE2-HEK293T cells for 24 h at 37 °C. Then, the medium was replaced with DMEM containing 10% FBS for 48 h. The ONE-Glo™ luciferase substrate (Promega; E6130) was used to generate luminescence signal (relative light units, RLU) that was detected with a spectrophotometer (SpectraMax^®^ iD3, Molecular Devices). The half maximal inhibitory concentration (IC50) was calculated using GraphPad Prism software.

### Immunosorbent assay

An immunosorbent assay was performed as described [24]. Plates were coated with 0.5 µg/ml of various SARS-CoV-2 S proteins or EpEx as negative control in 0.1 M NaHCO_3_ (pH 8.6) at 4 °C overnight. The variants of recombinant S protein were purchased from ACRO Biosystems (Alpha strain, SPN-C52H6; Beta strain, SPN-C52Hc; Gamma strain, SPN-C52Hg; Delta strain, SPN-C52He; Omicron BA.1, SPN-C5224; Omicron BA.2, SPN-C5223; Omicron BA.5, SPN-C522e). The B-S2-mAbs and normal mouse IgG (NMIgG; 100 ng/ml; Jackson ImmunoResearch; 015-000-003) were used as primary antibodies. The primary antibodies were added and incubated for 1 h at room temperature. Goat anti-mouse IgG conjugated horseradish peroxidase (HRP) (Jackson ImmunoResearch; 115-035-003) was used as the secondary antibody. HRP activity was detected using 3,3′5,5′-tetramethylbenzidine (TMB; ScyTek Laboratories; TM1999) substrate, as read with a spectrophotometer (SpectraMax; Molecular Devices) at OD450 nm.

### Immunofluorescence assay

Different strains of SARS-CoV-2 S protein were overexpressed in HET293T cells. The cells were fixed with 4% paraformaldehyde and stained with B-S2-mAbs or NMIgG (1 µg/ml) at 4 °C overnight. Then, the samples were incubated with FITC-conjugated anti-mouse IgG (Jackson ImmunoResearch; 115-095-166) and DAPI (Thermo Fisher Scientific; D3571). Images were acquired using an inverted microscope (Nikon ECLIPSE Ti2-U).

### Immunoblotting

Immunoblotting was performed as described [25]. Briefly, 100 ng recombinant S protein tagged with histidine residues was analyzed under reducing or non-reducing conditions. Variants of recombinant S protein of SARS-CoV-2 are described above. The samples were separated by SDS-PAGE and transferred to PVDF membranes (Millipore; Immobilon-P; IPVH00010), followed by hybridization with B-S2-mAbs (1 µg/ml; B-S2-mAb-2, -5, -7, -10, -13), normal mouse IgG (NMIgG; Jackson ImmunoResearch; 015-000-003), anti-His (GeneTex; GTX628914), and then secondary HRP-conjugated anti-mouse IgG antibody (Jackson ImmunoResearch; 115-035-003).

### Phage display biopanning

Phage display biopanning was performed as described [26, 27]. Briefly, Dynabeads protein G (Thermo Fisher Scientific;10004D) was conjugated with 1 µg B-S2-mAb-2 at 4 °C for 1 h. Then, 1 × 10^11^ pfu of phage-displayed peptide library (New England BioLabs; E8110S) was blocked with 1 µg NMIgG-conjugated Dynabeads protein G at 4 °C for 1 h. After blocking, the phage-displayed peptide library was incubated with B-S2-mAb-2-conjugated Dynabeads protein G and eluted by incubation with ER2738 *E. coli* (log phase). Eluted phages were then amplified in ER2738. The phage titer was counted by serial dilution on LB plates. The second and third biopanning rounds were performed with 1 × 10^11^ pfu amplified phages following the above steps. Single immune-positive phage clones were identified by immunosorbent assay as described [28].

## Results

### Generation of specific antibodies targeting the SARS-CoV-2 S2 subunit of S protein

**Fig. 1:**
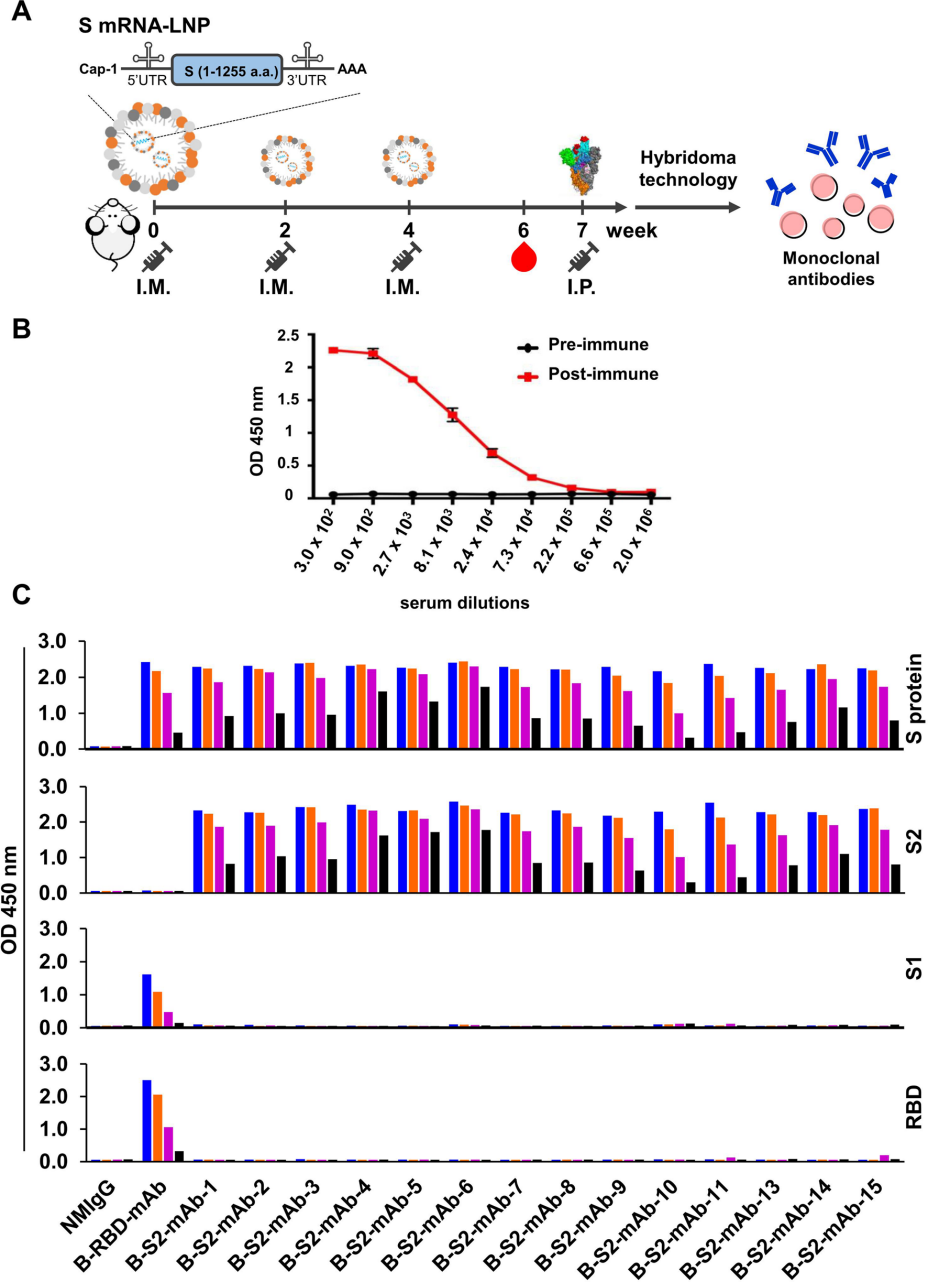
Generation of mAbs against the S2 subunit of SARS-CoV-2 S protein. **A** Schematic illustration of the immunization procedure. BALB/c mice were immunized by I.M. of 10 µg of mRNA-LNP encoding the Beta variant S protein every 2 weeks. A final booster of 25 µg trimeric Beta variant S protein was administered by I.P. on the seventh week. **B** Serum samples were harvested at week 6 and analyzed for antibody response to S protein. The immunosorbent assay indicated the antibody levels of post-immunized samples (red line) with serial dilutions compared with pre-immunized serum samples (black line). **C** The binding activities of fourteen serially diluted mAbs were detected by immunosorbent assay; plates were coated with recombinant full-length S protein (Beta strain), S1 (Wuhan strain), S2 (Wuhan strain) or RBD (Beta strain). Normal mouse IgG (NMIgG) was used as a negative control. B-RBD-mAb was used as a positive control for RBD and S1 subunit of SARS-CoV-2.

The continual evolution of SARS-CoV-2 raises major concerns about the possible emergence of variants that can escape current therapeutics due to mutations on the S1 subunit. We therefore sought to generate cross-reactive mAbs for betacoronaviruses by specifically targeting the S2 subunit. To do so, we used LNP-encapsulated mRNA encoding the S protein of Beta variant of SARS-CoV-2 for immunization of mice and boosted the animals with S-trimer protein to enhance the host immune responses. Specifically, BALB/c mice received I.M. injections of mRNA-LNP at weeks 0, 2 and 4, and a boosting dose of recombinant S protein via intraperitoneal injection (I.P.) at week 7 (Fig. 1A). Prior to the final booster dose, we analyzed the immune responses triggered by mRNA-LNP immunization. According to our immunosorbent assays, the antibody levels of S protein were dramatically increased after the third immunization (Fig. 1B). Finally, several mAbs were identified using mice hybridoma technology. Among the identified mAbs, fourteen recognized full-length S protein and the S2 subunit, but did not bind to the S1 subunit RBD domain (Fig. 1C). These antibodies were called B-S2-mAbs, as each displayed highly specific recognition for the S2 subunit of S protein.

### mAbs against the S2 subunit neutralize Gamma variant of SARS-CoV-2

**Fig. 2:**
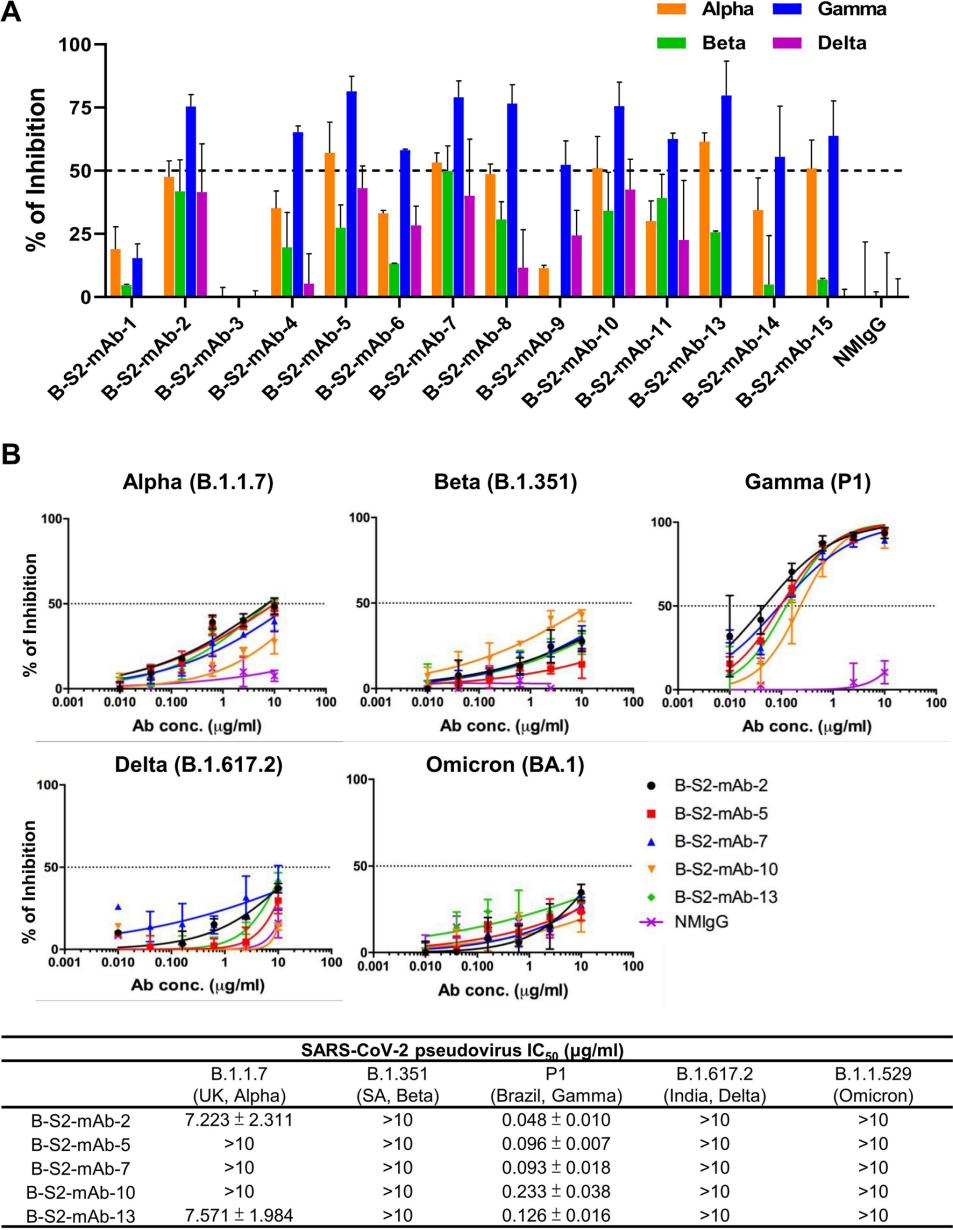
Neutralization of pseudotyped SARS-CoV-2 variants by anti-S2 mAbs. **A** Indicated B-S2-mAbs were assessed at 1 µg/ml in the neutralization assay, using the indicated VSV-based SARS-CoV-2 pseudoviruses. Each assay was performed in triplicate. **B** B-S2-mAb-2, -5, -7, -10, -13 were preincubated with SARS-CoV-2 Alpha, Beta, Gamma, Delta, Omicron BA.1 and BA.2 variant pseudoviruses. The IC_50_ values were calculated with GraphPad Prism software. Each assay was performed in triplicate, and all data points were used to calculate the mean ± SD. NMIgG was used as a negative control

To evaluate whether the S2-specific mAbs exhibit broad-spectrum neutralization functions toward SARS-CoV-2 variants, we performed two independent in vitro VSV-based pseudovirus neutralization screens with numerous variants. In the first screen, twelve out of fourteen tested B-S2-mAbs were above the 50% inhibitory concentration (IC_50_) when applied at 1 µg/ml to the Gamma variant, but not Alpha, Beta, Delta and Omicron BA.1 pseudotyped viruses (Fig. 2A; Additional file 1: Fig. S1). Among the tested mAbs, B-S2-mAbs- 2, -5, -7, -10 and -13 displayed the most effective inhibition of the pseudotyped Gamma variant. We further examined the neutralization ability of these five mAbs by mixing serial dilutions with other variants, including Alpha, Beta, Gamma, Delta and Omicron BA.1. Consistent with the previous results, the VSV-based pseudovirus neutralization assays revealed that each of the five B-S2-mAbs exhibited reliable neutralization potency against Gamma variant (IC_50_ values ranged from 0.048 to 0.233 µg/ml), but the B-S2-mAbs had poor activities against other variants (Fig. 2B). Compared with the Wuhan strain, the Gamma variant carries two mutations (T1027I and V1176F) in the S2 subunit and 10 mutations in the S1 subunit; this variant caused a major two-wave infection in Brazil during 2021 [29]. Together our in vitro neutralization assay results suggested that B-S2-mAb-2, -5, -7, -10 and -13 display good neutralization potencies against the Gamma variant of SARS-CoV-2.

### B-S2-mAbs broadly bind to variants of SARS-CoV-2

**Fig. 3:**
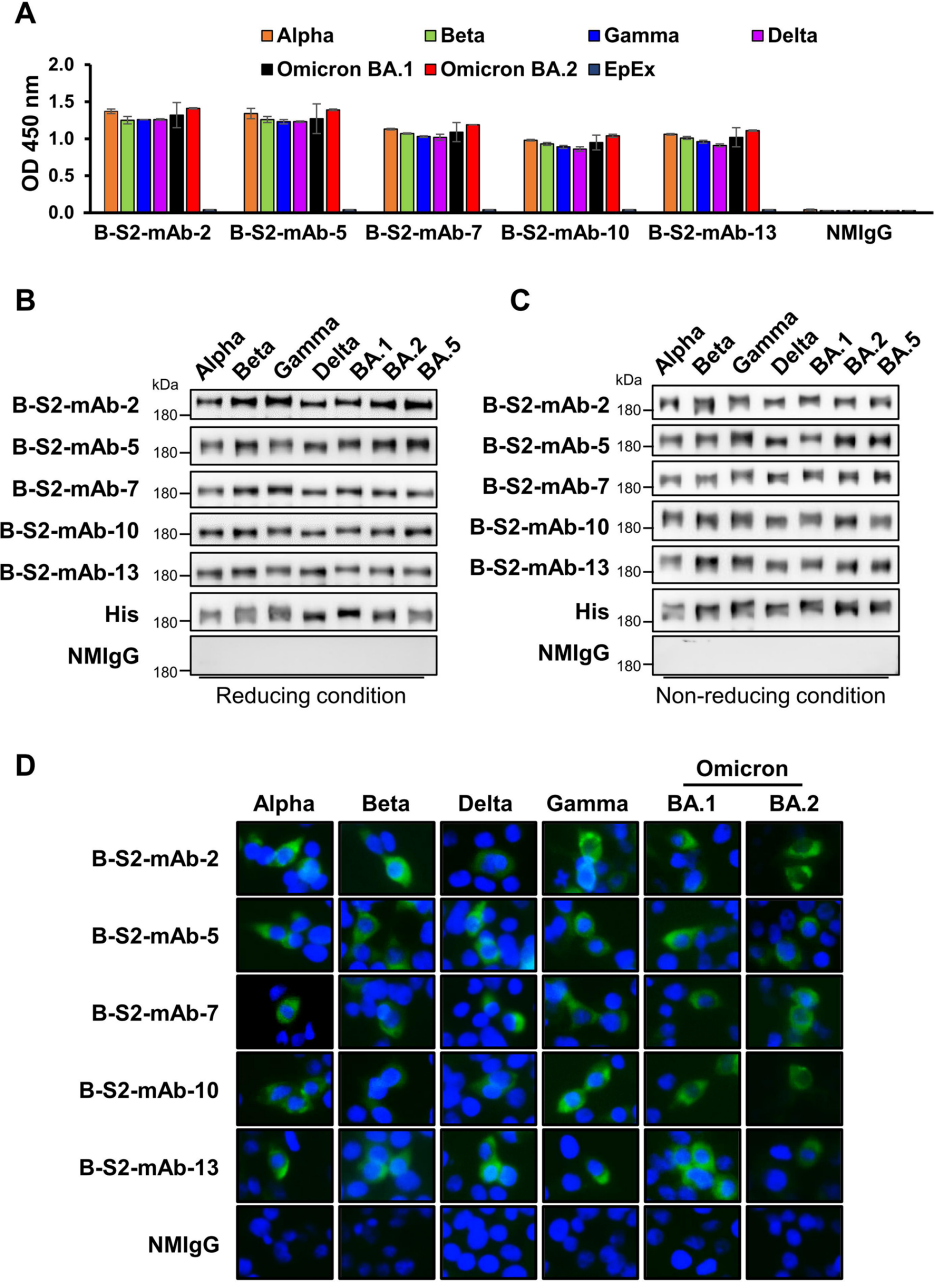
S2 antibodies recognize S proteins of SARS-CoV-2 variants. **A** Comparative ELISA was performed by coating plates with 0.5 µg/ml recombinant S protein of indicated SARS-CoV-2 variants and incubating with B-S2-mAb-2, -5, -7, -10, -13 (100 ng/ml). EpEx and NMIgG served as negative controls. **B** The five B-S2-mAbs (1 µg/ml) were used as primary antibodies to detect recombinant S proteins of SARS-CoV-2 Alpha, Beta, Gamma, Delta, Omicron BA.1, BA.2 and BA.5 variants by immunoblotting under reducing conditions. **C** Immunoblotting under non-reducing conditions was performed to detect variants of tagged-recombinant S protein by indicated B-S2-mAbs (used at 1 µg/ml). In panels B and C, His mAb was used as a positive and loading control. **D** Immunofluorescence assay to detect overexpressed S protein of the indicated SARS-CoV-2 variants in HEK293T cells. The assay was performed using the five B-S2-mAbs (1 µg/ml). NMIgG served as a negative control

In our further characterization of these mAbs, we found that 80% of the B-S2-mAbs were mouse IgG1/Kappa isotype (Table 1). In addition, we performed several functional assays to examine the cross-reactivity potentials for S proteins of prominent SARS-CoV-2 variants. The binding abilities were assessed using an immunosorbent assay in which trimeric S protein was coated on plates and incubated with serially diluted B-S2-mAbs. We found that B-S2-mAb-2, -5, -7, -10 and -13 exhibited high-affinity binding to the S-trimers of Alpha, Beta, Gamma, Delta and Omicron sublineages BA.1 and BA.2 (Fig. 3A). Since the Omicron sublineage BA.5 is quickly becoming the dominant variant around the world, we performed comparative immunoblotting experiments with the Omicron BA.5 S proteins and other variants. The anti-His mAb can recognize tagged recombinant proteins and was used as an internal control to detect S protein variant loading amounts (Fig. 3B, C). The five B-S2-mAbs recognized all of the S proteins of SARS-CoV-2 variants (including Omicron BA.1, BA.2 and BA.5), according to immunoblotting assays performed under reducing and non-reducing conditions (Fig. 3B, C). Next, we overexpressed a variety of S proteins in HEK293T cells and observed that these five B-S2-mAbs successful hybridized to the S protein-expressing cells in immunofluorescence assays (Fig. 3D). Overall, our study yielded five mAbs (i.e., B-S2-mAb-2, -5, -7, -10 and -13) that can recognize S proteins with trimeric or linear structure derived from multiple SARS-CoV-2 variants, including Alpha, Beta, Gamma, Delta and Omicron sublineages (Table 1). Our results suggested these B-S2-mAbs may be useful in widespread applications, such as immunosorbent, immunoblotting and immunofluorescence assays.

**Table 1:** Characterization of B-S2-mAbs by ELISA, WB, IFA and neutralization IC_50_

mAb/isotype	SARS-CoV-2	ELISA	WB	IFA	IC_50_ (µg/ml)
B-S2-mAb-1/IgG1, κ	Wuhan	** + **	N.D	N.D	N.D
	Alpha	N.D	N.D	N.D	N.D
	Beta	** + **	N.D	N.D	N.D
	Gamma	N.D	N.D	N.D	N.D
	Delta	N.D	N.D	N.D	N.D
	Omicron BA.1	N.D	N.D	N.D	N.D
	Omicron BA.2	N.D	N.D	N.D	N.D
	Omicron BA.5	N.D	N.D	N.D	N.D
B-S2-mAb-2/IgG1, κ	Wuhan	** + **	N.D	N.D	N.D
	Alpha	** + **	** + **	** + **	7.223 ± 2.311
	Beta	** + **	** + **	** + **	> 10
	Gamma	** + **	** + **	** + **	0.048 ± 0.010
	Delta	** + **	** + **	** + **	> 10
	Omicron BA.1	** + **	** + **	** + **	> 10
	Omicron BA.2	** + **	** + **	** + **	N.D
	Omicron BA.5	N.D	** + **	N.D	N.D
B-S2-mAb-3/IgG1, κ	Wuhan	** + **	N.D	N.D	N.D
	Alpha	N.D	N.D	N.D	N.D
	Beta	** + **	N.D	N.D	N.D
	Gamma	N.D	N.D	N.D	N.D
	Delta	N.D	N.D	N.D	N.D
	Omicron BA.1	N.D	N.D	N.D	N.D
	Omicron BA.2	N.D	N.D	N.D	N.D
	Omicron BA.5	N.D	N.D	N.D	N.D
B-S2-mAb-4/IgG1, κ	Wuhan	** + **	N.D	N.D	N.D
	Alpha	N.D	N.D	N.D	N.D
	Beta	** + **	N.D	N.D	N.D
	Gamma	N.D	N.D	N.D	N.D
	Delta	N.D	N.D	N.D	N.D
	Omicron BA.1	N.D	N.D	N.D	N.D
	Omicron BA.2	N.D	N.D	N.D	N.D
	Omicron BA.5	N.D	N.D	N.D	N.D
B-S2-mAb-5/IgG1, κ	Wuhan	** + **	N.D	N.D	N.D
	Alpha	** + **	** + **	** + **	> 10
	Beta	** + **	** + **	** + **	> 10
	Gamma	** + **	** + **	** + **	0.096 ± 0.007
	Delta	** + **	** + **	** + **	> 10
	Omicron BA.1	** + **	** + **	** + **	> 10
	Omicron BA.2	** + **	** + **	** + **	N.D
	Omicron BA.5	N.D	** + **	N.D	N.D
B-S2-mAb-6/IgG2b, κ	Wuhan	** + **	N.D	N.D	N.D
	Alpha	N.D	N.D	N.D	N.D
	Beta	** + **	N.D	N.D	N.D
	Gamma	N.D	N.D	N.D	N.D
	Delta	N.D	N.D	N.D	N.D
	Omicron BA.1	N.D	N.D	N.D	N.D
	Omicron BA.2	N.D	N.D	N.D	N.D
	Omicron BA.5	N.D	N.D	N.D	N.D
B-S2-mAb-7/IgG1, κ	Wuhan	** + **	N.D	N.D	N.D
	Alpha	** + **	** + **	** + **	> 10
	Beta	** + **	** + **	** + **	> 10
	Gamma	** + **	** + **	** + **	0.093 ± 0.018
	Delta	** + **	** + **	** + **	> 10
	Omicron BA.1	** + **	** + **	** + **	> 10
	Omicron BA.2	** + **	** + **	** + **	N.D
	Omicron BA.5	N.D	** + **	N.D	N.D
B-S2-mAb-8/IgG1, κ	Wuhan	** + **	N.D	N.D	N.D
	Alpha	N.D	N.D	N.D	N.D
	Beta	** + **	N.D	N.D	N.D
	Gamma	N.D	N.D	N.D	N.D
	Delta	N.D	N.D	N.D	N.D
	Omicron BA.1	N.D	N.D	N.D	N.D
	Omicron BA.2	N.D	N.D	N.D	N.D
	Omicron BA.5	N.D	N.D	N.D	N.D
B-S2-mAb-9/IgG1, κ	Wuhan	** + **	N.D	N.D	N.D
	Alpha	N.D	N.D	N.D	N.D
	Beta	** + **	N.D	N.D	N.D
	Gamma	N.D	N.D	N.D	N.D
	Delta	N.D	N.D	N.D	N.D
	Omicron BA.1	N.D	N.D	N.D	N.D
	Omicron BA.2	N.D	N.D	N.D	N.D
	Omicron BA.5	N.D	N.D	N.D	N.D
B-S2-mAb-10/IgG1, κ	Wuhan	** + **	N.D	N.D	N.D
	Alpha	** + **	** + **	** + **	> 10
	Beta	** + **	** + **	** + **	> 10
	Gamma	** + **	** + **	** + **	0.233 ± 0.038
	Delta	** + **	** + **	** + **	> 10
	Omicron BA.1	** + **	** + **	** + **	> 10
	Omicron BA.2	** + **	** + **	** + **	N.D
	Omicron BA.5	N.D	** + **	N.D	N.D
B-S2-mAb-11/IgG2a, κ	Wuhan	** + **	N.D	N.D	N.D
	Alpha	N.D	N.D	N.D	N.D
	Beta	** + **	N.D	N.D	N.D
	Gamma	N.D	N.D	N.D	N.D
	Delta	N.D	N.D	N.D	N.D
	Omicron BA.1	N.D	N.D	N.D	N.D
	Omicron BA.2	N.D	N.D	N.D	N.D
	Omicron BA.5	N.D	N.D	N.D	N.D
B-S2-mAb-13/IgG1, κ	Wuhan	** + **	N.D	N.D	N.D
	Alpha	** + **	** + **	** + **	7.571 ± 1.984
	Beta	** + **	** + **	** + **	> 10
	Gamma	** + **	** + **	** + **	0.126 ± 0.016
	Delta	** + **	** + **	** + **	> 10
	Omicron BA.1	** + **	** + **	** + **	> 10
	Omicron BA.2	** + **	** + **	** + **	N.D
	Omicron BA.5	N.D	** + **	N.D	N.D
B-S2-mAb-14/IgG1, κ	Wuhan	** + **	N.D	N.D	N.D
	Alpha	N.D	N.D	N.D	N.D
	Beta	** + **	N.D	N.D	N.D
	Gamma	N.D	N.D	N.D	N.D
	Delta	N.D	N.D	N.D	N.D
	Omicron BA.1	N.D	N.D	N.D	N.D
	Omicron BA.2	N.D	N.D	N.D	N.D
	Omicron BA.5	N.D	N.D	N.D	N.D
B-S2-mAb-15/IgG2a, κ	Wuhan	** + **	N.D	N.D	N.D
	Alpha	N.D	N.D	N.D	N.D
	Beta	** + **	N.D	N.D	N.D
	Gamma	N.D	N.D	N.D	N.D
	Delta	N.D	N.D	N.D	N.D
	Omicron BA.1	N.D	N.D	N.D	N.D
	Omicron BA.2	N.D	N.D	N.D	N.D
	Omicron BA.5	N.D	N.D	N.D	N.D

+ positive results, − negative results, *N.D.* not determined

### Identification of B-S2-mAb-2 neutralizing epitope

**Fig. 4:**
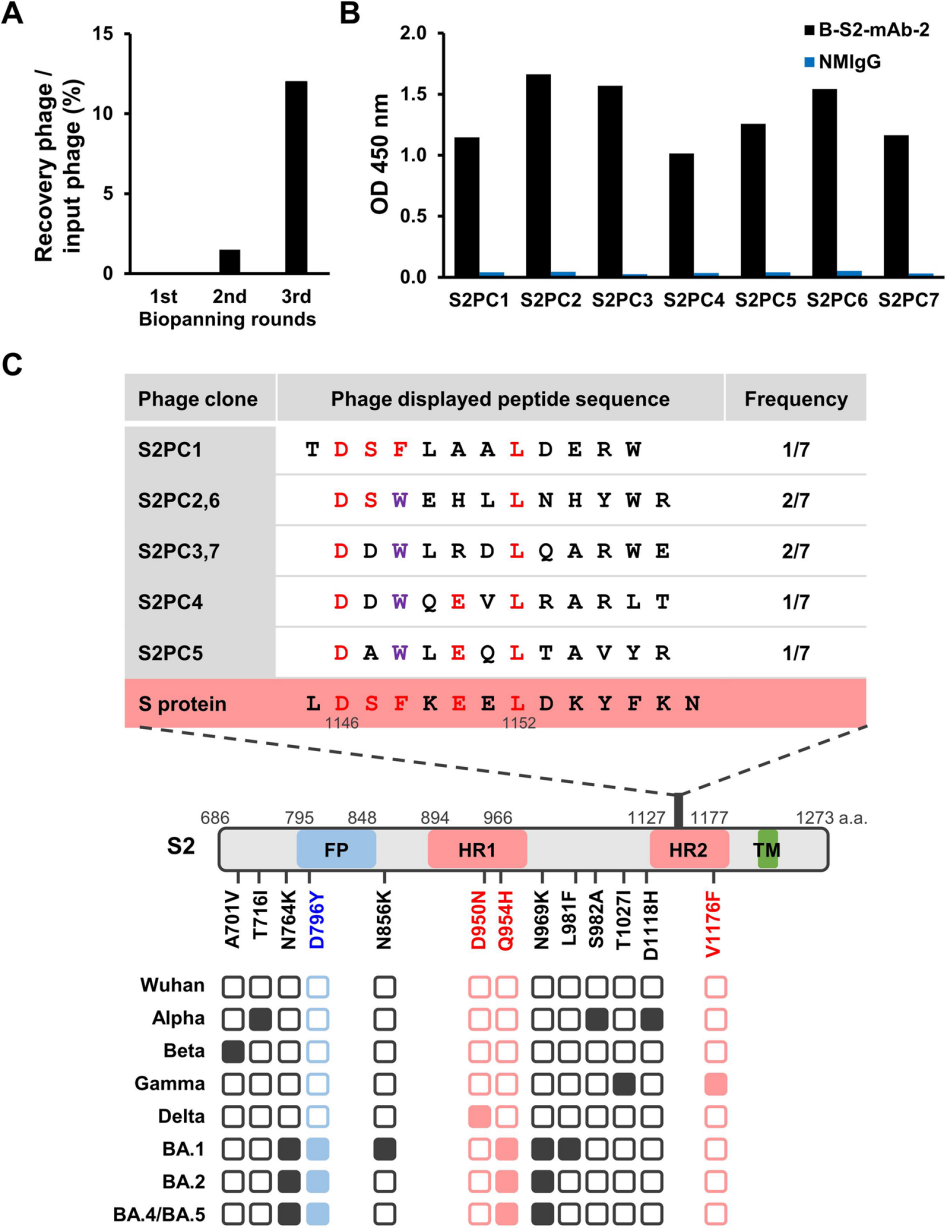
Epitope mapping of B-S2-mAb-2 with a phage-displayed peptide library. **A** The recovery ratios are shown for three rounds of biopanning. The input phage indicates the reacted titer of peptide-fused phages. The recovery phage indicates the eluted titer of peptide-fused phages interacting with B-S2-mAb-2. **B** Immunosorbent assay was performed to evaluate the binding of phage clones with B-S2-mAb-2. B-S2-mAb-2 (1 µg/ml) was coated on the ELISA plate. Normal mouse IgG (NMIgG) was used as a negative control. **C** The phage clones were analyzed. Red color indicates consensus amino acids. Tryptophan (W; purple) and phenylalanine (F) amino acids are structurally related aromatic amino acids. Illustration shows the domains of the S2 subunit (FP, fusion peptide; HR1, heptad repeats 1; HR2, heptad repeats; TM, transmembrane) and the occurrence of mutations in SARS-CoV-2 variants (Wuhan, Alpha, Beta, Gamma, Delta, Omicron BA.1, Omicron BA.2, Omicron BA.4/BA.5). Empty box indicates no mutation compared to the original Wuhan strain

The peptide mapping experiment utilized twelve phage-displayed random-peptide libararies to isolate peptides that interact with B-S2-mAb-2. This mAb was used in the experiment because it displayed the most effective neutralizing activity. After three biopanning rounds, the eluted phage titers were increased 400-fold compared to the initital elution titer (Fig. 4A). Seven phage clones were isolated after the third round of biopanning and exhibited significant reactivity to B-S2-mAb-2 (Fig. 4B). Alignment of the seven peptides displayed by the selected phage clones revealed a consensus motif of D-S-F/W-x-E-x-L (x represents any amino acid) (Fig. 4C). On the basis of this consensus sequence, a neutralizing epitope of B-S2-mAb-2, D_1146_SFKEEL_1152_, was located in the S2 subunit HR2 domain (Fig. 4C). Importantly, this motif (amino acid residues 1146–1152) has been strictly conserved during the evolution of SARS-CoV-2. These results can explain why B-S2-mAb-2 can be broadly applied in serveal functional assays to recognize S protein of multiple SARS-CoV-2 variants.

## Discussion

Most of the SARS-CoV-2 neutralizing antibody therapeutics and vaccines currently in use have been developed by targeting the RBD; however, this region has undergone dramatic changes during the evolution of SARS-CoV-2 variants [7]. Mutations within the N-terminal domain (NTD) and RBD of SARS-CoV-2 S1 subunit have had substantial negative effects on the neutralizing efficacies of many therapeutics. For example, the therapeutic antibody sotrovimab (GlaxoSmithKline and Vir Biotechnology) and antibody cocktails of casirivimab/imdevimab (Regeneron Pharmaceuticals) and bamlanivimab/etesevimab (Eli Lilly and Company) can protect against several SARS-CoV-2 variants, but have little effect on Omicron BA.2. The immune escape of Omicron BA.2 is thought to be due to its numerous mutations in the NTD and RBD. These mutations have greatly reduced the neutralizing activities of the above-mentioned therapeutic antibodies, leading the U.S. FDA to pause their use for treatment of COVID-19 [30, 31].

In contrast, SARS-CoV-2 variants rarely accumulate mutations in the S2 subunit [32]. Nevertheless, Delta and Omicron variants both carry D950N and Q954H mutations in the HR1 domain, which might affect the interactions between HR1 and HR2 domains that participate in six-helical bundle formation for viral fusion and entry. Importantly, the amino acid residues 816–843 of the FP and 1140–1161 of HR2 are highly conserved across SARS-CoV-2 variants and also conserved in other betacoronaviruses, such as SARS-CoV, MERS-CoV, HCoV-OC43 and HCoV-HKU1 [33]. Using phage display, we identified a neutralizing epitope of B-S2-mAb-2 that is also located at 1146–1152 of the HR2 domain in S protein. The neutralizing antibodies, CV3-25, S2P6 and CC40.8, which were isolated from COVID-19 convalescent donors, target the HR2 domain to inhibit membrane fusion [15, 16, 34]. While these antibodies have high measured IC_50_ values in pseudovirus inhibition assays, the therapeutics are more effective in vivo, suggesting that Fc-mediated effector functions may be important to reduce viral burden [15, 34]. In addition, the COV44-62 and COVI44-79 antibodies target the FP domain of the S2 subunit and partially neutralize variants of SARS-CoV-2, along with their optimal Fc effector function observed in hamster models of disease [18]. Based on these studies and ours, it is likely that mAbs with optimized Fc regions that target the FP or HR2 domains of the S2 subunit may exhibit broad neutralizing activity against multiple betacoronaviruses.

In this work, we generated several mAbs that specifically target the S2 subunit of SARS-CoV-2 variants using an mRNA-LNP strategy of immunization. Among these antibodies, we identified five that recognize different viral S protein variants, including Alpha, Beta, Gamma, Delta, and Omicron (BA.1, BA.2 and BA.5). These antibodies may be useful in widespread applications, including immunosorbent, immunofluorescence assay and immunoblotting assays (Fig. 3; Table 1). Moreover, the S2 subunit is a conserved region that participates in the membrane fusion of betacoronaviruses. The five B-S2-mAbs had suitable neutralizing activities against the Gamma strain of SARS-CoV-2, with low IC_50_ values ranging from 0.048 µg/ml to 0.126 µg/ml (Fig. 2; Table 1). The SARS-CoV-2 Gamma strain is refractory to neutralization by several neutralizing Abs, including casirivimab, bamlanivimab and etesevimab [7]. The Gamma variant carries T1027I and V1176F mutants on the S2 subunit that may cause a conformation change in the stalk that facilitates B-S2-mAbs binding and neutralization. Furthermore, we demonstrated that B-S2-mAb-2 binds to residues 1146–1152 of HR2 domain in S protein, which is conserved not only in SARS-CoV-2 variants but also in other betacoronaviruses. Such a high level of conservation suggests the targeting of this domain can lead to broad recognition (Fig. 4). Therefore, these antibodies may serve as starting points for the development of novel therapeutics to be used in the current and future pandemics.

## Conclusion

The coronavirus S protein has undergone many evolutionary changes, but the viral S2 subunit retains several domains that are highly conserved among betacoronaviruses. Nevertheless, S2 subunit-targeting mAbs are not as well developed or as widely discussed as RBD-neutralizing antibodies, largely because they affect viral fusion but do not directly inhibit viral entry. Here, we identified five mAbs that specifically target the S2 subunit and exhibit high binding ability across SARS-CoV-2 variants. Although these five B-S2-mAbs displayed neutralizing abilities restricted to the Gamma strain, each exhibited broad recognition of S proteins from most SARS-CoV-2 variants, including Omicron sublineages. Therefore, our set of B-S2-mAbs may potentially be utilized for broad coronavirus detection and selective neutralization in basic research and clinical applications.

## Supplementary information

**Additional file 1**: **Fig. S1** Neutralization activity of B-S2-mAbs against pseudotyped Omicron BA.1. Indicated B-S2-mAbs were assessed at 1 µg/ml in a pseudotyped neutralization assay with Omicron BA.1 variant. Each assay was performed in triplicate.

## Data Availability

The datasets analyzed in the current study are available upon reasonable request.

## Abbreviations

COVID-19: Coronavirus disease 2019
SARS-CoV-2: Severe acute respiratory syndrome coronavirus 2
NTD: N-terminal domain
RBD: Receptor-binding domain
hACE2: Human angiotensin converting enzyme 2
IC_50_: Half maximal inhibitory concentration
mAb: Monoclonal antibody
FP: Fusion peptide
HR1: Heptad repeats 1
HR2: Heptad repeats 2
