# Supplementary material for: Monoclonal antibodies against S2 subunit of spike protein exhibit broad reactivity toward SARS-CoV-2 variants
Source: J Biomed Sci. 2022 Dec 22;29:108. doi: 10.1186/s12929-022-00891-2 (PMC9774083; doi:10.1186/s12929-022-00891-2)
Supplement: Supplementary file 1 — Additional file 1: Fig. S1 Neutralization activity of B-S2-mAbs against pseudotyped Omicron BA.1. Indicated B-S2-mAbs were assessed at 1 µg/ml in a pseudotyped neutralization assay with Omicron BA.1 variant. Each assay was performed in triplicate. [file 12929_2022_891_MOESM1_ESM.docx]

**Additional file 1**

**Monoclonal antibodies against** **S2 subunit of spike protein exhibit broad reactivity toward SARS-CoV-2 variants**

Shih-Han Ko^1^, Wan-Yu Chen^2^, Shih-Chieh Su^2^, Hsiu-Ting Lin^2^, Feng-Yi Ke^1^, Kang-Hao Liang^1^, Fu-Fei Hsu^1^, Monika Kumari^2^, Chi-Yu Fu^2^, and Han-Chung Wu^1,2, *^

^1^Biomedical Translation Research Center (BioTReC), Academia Sinica, Taipei, 11529, Taiwan

^2^Institute of Cellular and Organismic Biology, Academia Sinica, Taipei, 11529, Taiwan

**Supplementary Figure**


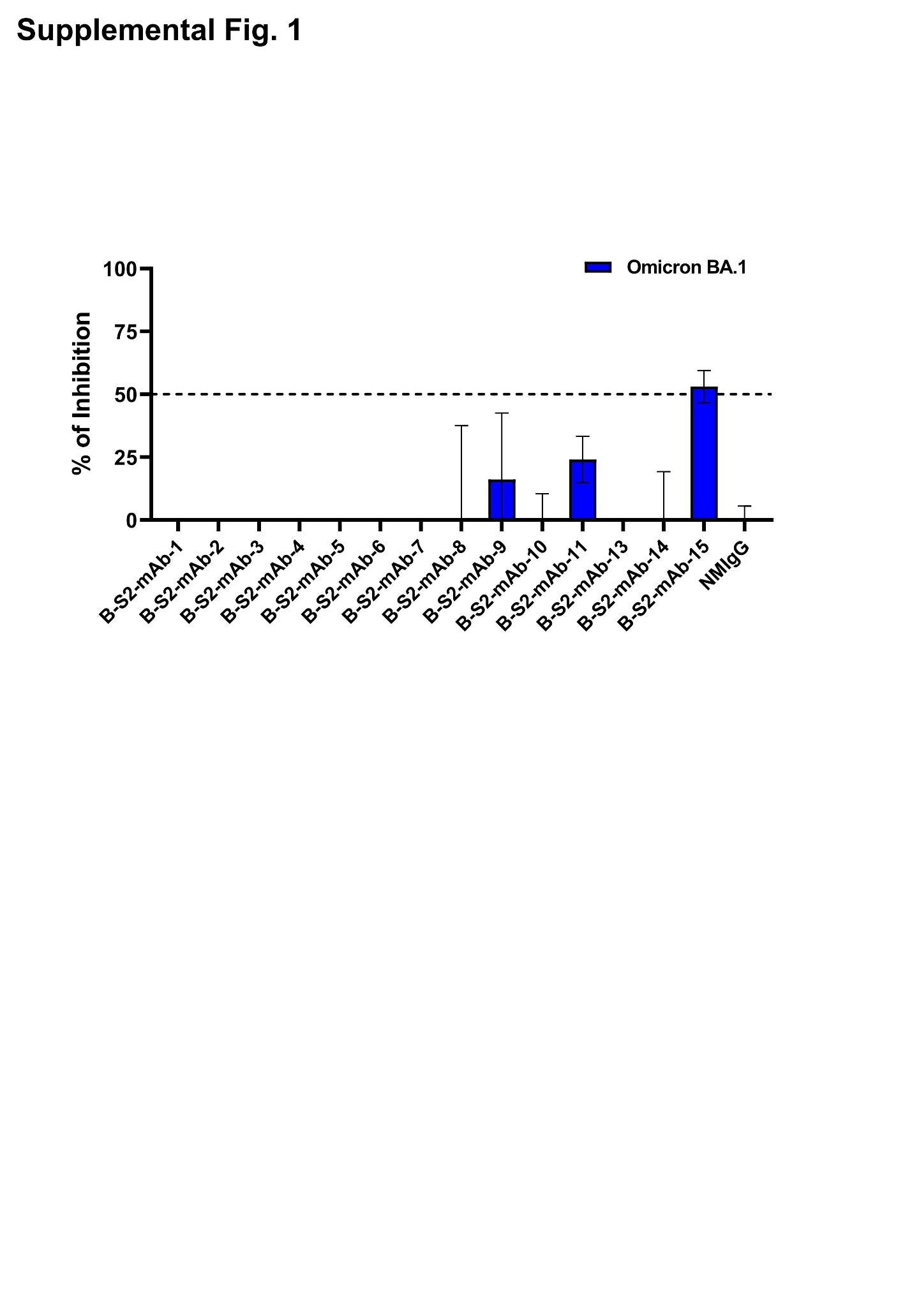


**Fig. S1** Neutralization activity of B-S2-mAbs against pseudotyped Omicron BA.1. Indicated B-S2-mAbs were assessed at 1 µg/ml in a pseudotyped neutralization assay with Omicron BA.1 variant. Each assay was performed in triplicate.
